# Effect of statins on fasting glucose in non-diabetic individuals: nationwide population-based health examination in Korea

**DOI:** 10.1186/s12933-018-0799-4

**Published:** 2018-12-05

**Authors:** Jinkwon Kim, Hye Sun Lee, Kyung-Yul Lee

**Affiliations:** 1 Department of Neurology, CHA Bundang Medical Center, CHA University College of Medicine, Seongnam, Republic of Korea; 2 Biostatistics Collaboration Unit, Yonsei University College of Medicine, Seoul, Republic of Korea; 3 Department of Neurology, Gangnam Severance Hospital, Yonsei University College of Medicine, 211 Eonju-ro, Gangnam-gu, Seoul, 06273 Republic of Korea

**Keywords:** Statin, Glucose, Diabetes mellitus, Health examination

## Abstract

**Background:**

Increasing evidence suggest that statin therapy has a diabetogenic effect. Individual types of statin may have a different effect on glucose metabolism. Using the repeated nationwide population-based health screening data in Korea, we investigated the longitudinal changes in fasting glucose level of non-diabetic individuals by use of statins.

**Methods:**

From the National Health Screening Cohort, we included 379,865 non-diabetic individuals who had ≥ 2 health screening examinations with fasting blood glucose level measured in 2002–2013. Using the prescription records of statins in the database, we calculated the proportion of days covered (PDC) and average number of defined daily doses per day (anDDD) by statins. We constructed multivariate linear mixed models to evaluate the effects of statins on the changes in fasting glucose (Δglu).

**Results:**

High PDC by statins had a significant positive effect on Δglu (coefficient for PDC 0.093 mmol/L, standard error 0.007, p < 0.001). anDDD by statins was also positively associated with Δglu (coefficient for anDDD 0.119 mmol/L, standard error 0.009, p < 0.001). Unlike statins, the PDC by fibrate and ezetimibe were not significantly associated with Δglu. There was no significant interaction effect on Δglu between time interval and statin. Considering individual types of statins, use of atorvastatin, rosuvastatin, pitavastatin, and simvastatin were significantly associated with increase of Δglu. Pravastatin, lovastatin, and fluvastatin were also positively associated with Δglu, but were not statistically significant.

**Conclusions:**

More adherent and intensive use of statins was significantly associated with an increase in fasting glucose of non-diabetic individuals. In subgroup analysis of individual statins, use of atorvastatin, rosuvastatin, pitavastatin and simvastatin had significant association with increase in fasting glucose. Pravastatin, lovastatin, and fluvastatin had non-significant trend toward an increased fasting glucose. Our findings suggest the medication class effect of statins inducing hyperglycemia.

## Background

Statins, 3-hydroxy-3-methylglutaryl-coenzyme reductase (HMGCR) inhibitors, are class of lipid lowering medications with pleiotropic properties due to anti-inflammatory, anti-thrombotic, and anti-oxidative effects [1]. It is well-established that statins can reduce the risk for cardiovascular and cerebrovascular diseases [2]. In clinical practice, statins have a major role in the prevention of cardiovascular disease for those at higher risk. Besides the cardiovascular benefits, there are concerns that statins may lead to hyperglycemia and increase the risk of new-onset diabetes mellitus (DM) [3–5]. In randomized and observational studies, there was a higher incidence of new-onset DM in patients receiving statin compared to those who were not [4]. As DM is a major risk factor of cardiovascular disease and various long-term complications, this is a clinically important issue. But also DM, impaired fasting glucose and pre-diabetes are significant cardiovascular risk factor [6–8]. Experimental and clinical data suggested that statins may lead to the increase of insulin-resistance and hyperglycemia [9, 10]. However, there are still insufficient data for the longitudinal changes in fasting glucose with the use of statins. In addition, there are multiple types of statin, each with a different chemical structure and pharmacokinetic profile [11, 12]. The individual statin types may have different effects on glucose metabolism [13, 14]. To investigate these issues, we conducted a longitudinal study to analyze the changes in fasting glucose by use of statins based on data from repeatedly performed nationwide health examinations in Korea.

## Methods

### Data source

This study was based on the National Health Insurance Service-National Health Screening Cohort (NHIS-HEALS) in Korea [15]. The NHIS offers a free health examination program to all members ≥ 40 years old every 2 years. Among the Korean subjects aged 40–79 who underwent the health examination during 2002–2003, about 10% (n = 541,866) were randomly selected to form the NHIS-HEALS. NHIS-HEALS contained serial health examination data including fasting glucose, blood pressure, body mass index (BMI), questionnaire for lifestyle, and every individual’s health insurance claim data for hospital visits, medical procedures, diagnoses (based on International Statistical Classification of Diseases and Related Health Problems 10th Revision, ICD-10), and drug prescriptions during the study period of 2002–2013. All included subjects were followed-up until death, loss of eligibility for NHIS due to emigration, or Dec 31, 2013, which is the end of the study period.

### Study subjects

To investigate the longitudinal change in fasting glucose, we selected subjects who had data on their fasting glucose level from at least 2 health examinations during the study period of 2002–2013. For each subject, the baseline value of fasting glucose was defined as the value at the first health examination. As the use of anti-diabetic medication can influence the fasting glucose level, we only included non-diabetic individuals. Therefore, we excluded patients who (1) had a fasting glucose level ≥ 7 mmol/L at the baseline health examination, (2) answered “yes” to the diagnosis of DM on the questionnaire in health examination, (3) had a diagnosis of DM (ICD-10 code ‘E10–E15’) from the health insurance claim data, and (4) had received a prescription for anti-diabetic medication during the study period.

### Data collection

In the serial NHIS health examinations, data were collected for sex, age, BMI, household income, and systolic blood pressure. At each examination, fasting glucose was measured with a venous blood sample after more than 6 h of fasting. The questionnaire for lifestyle in the health examination contained questions regarding smoking status, alcohol consumption, and physical activity [15]. Alcohol consumption was classified into ‘less than 1 time’, ‘1–2 times’, ‘3–4 times’, and ‘≥ 5 times’ according to the how many times alcohol was consumed per week on average. Physical activity was grouped into ‘< 1 day’, ‘1–4 days’, and ‘≥ 5 days’ according to the number of days of exercise per week on average (regardless of type or duration of exercise). Health examination data with missing values for any of fasting glucose or covariates (sex, age, BMI, household income, systolic blood pressure, smoking status, alcohol consumption, and physical activity) were excluded from the analysis. Health examination data with extreme values of fasting glucose (≤ 3 or ≥ 25 mmol/L) were also excluded [16]. If the initial health examination data in each patient was excluded due to incomplete data for fasting glucose or covariates, the next health examination was treated as baseline data for the patient.

### Statistical analysis with data for medications

As fasting glucose was measured repeatedly, we constructed a linear mixed-effect model to analyze the change in fasting glucose (Δglu = glu_exam_ − glu_base_, mmol/L) over time. In the linear mixed-effect model, variables of fixed effects were sex, age, fasting glucose level at baseline (initial measurement), time interval (Δtime; time between baseline and serial measurements), use of statins during the time interval, and other clinical characteristics obtained from the serial NHIS health examinations (systolic blood pressure, BMI, household income, smoking status, alcohol consumption, and physical activity). The general structure for the mixed model was Δglu = β*time + β*statin + β*other covariates + *intercept* (where β is a regression coefficient for the variable). To evaluate the effect of statins on the change in fasting glucose, we calculated the β of statins in the linear mixed model. As markers of statin use during the period between baseline and serial measurements, we calculated 1) proportion of days covered (PDC) = ‘number of days covered by statins divided by total number of days’ and 2) average number of defined daily doses per day (anDDD) = ‘cumulative doses of statins during the period divided by total number of days and defined daily dose of each statin’. Defined daily doses (DDD) of each statin were 20 mg for atorvastatin, 10 mg for rosuvastatin, 2 mg for pitavastatin, 30 mg for pravastatin, 30 mg for simvastatin, 45 mg for lovastatin, and 60 mg for fluvastatin according to the World Health Organization. If a subject received 40 mg of atorvastatin for 180 days during a period of 760 days between the baseline and next NHIS health examination, PDC by statins over the period would be 180/760 = 0.25 and anDDD by statins would be 40*180/20/760 = 0.5. If a subject received 20 mg of atorvastatin for 60 days followed by 10 mg of rosuvastatin for 120 days during a period of 360 days between the baseline and next NHIS health examination, PDC by statins would be (60 + 120)/360 = 0.5 (PDC by atorvastatin would be 0.166 and PDC by rosuvastatin would be 0.333) and anDDD by statins would be (20*60/20 + 10*120/10)/360 = 0.570. In this case, anDDD by atorvastatin would be 20*60/20/360 = 0.237 and anDDD by rosuvastatin would be 10*120/10/360 = 0.333. To evaluate the effects of non-statin lipid-lowering medications on glucose, we also calculated data of PDC by fibrate (bezafibrate, ciprofibrate, etofibrate, fenofibrate, and gemfibrozil) and ezetimibe during the study period in the same manner. The data management and statistical analyses were performed with the SAS statistical software package, version 9.4 (SAS Institute Inc., Cary, NC, USA) and R software, version 3.4.3 (The R Foundation for Statistical Computing, Vienna, Austria; http://www.R-project.org/). A two-sided p < 0.05 was regarded as statistically significant.

## Results

### Subjects

**Fig. 1:**
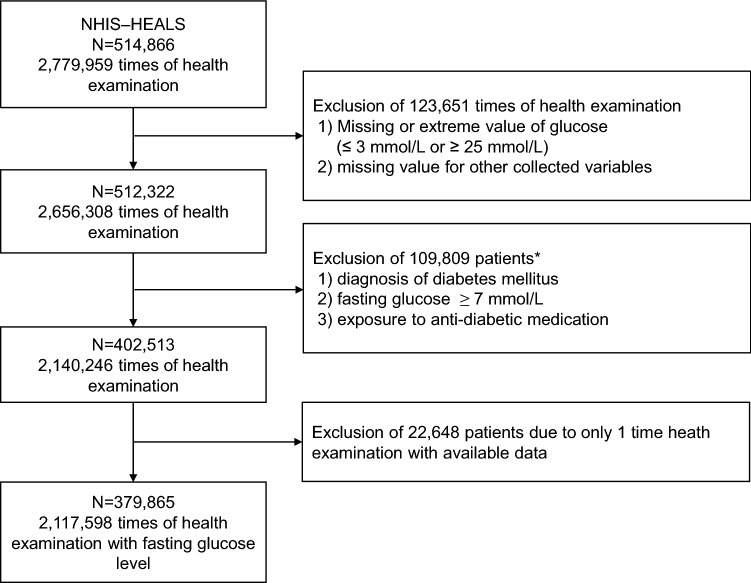
Flow chart of patients according to inclusion and exclusion criteria. NHIS-HEALS, the National Health Insurance Service-National Health Screening Cohort in Korea. *Those who met the exclusion criteria in any time of study period were excluded

According to the inclusion and exclusion criteria, we finally included 379,865 non-diabetic subjects who received ≥ 2 measurements of fasting glucose level (Fig. 1). The mean age at the baseline NHIS health examination was 51.9 ± 9.2 years and males comprised 52.6% of the subjects. During the study period, the median number of glucose measurements per subject was 5 (interquartile range, 4–6). The clinical characteristics of the included subjects are summarized in Table 1. Among the included subjects, the prevalence of exposure to statins (at least 1 prescription of statins during the study period) was 25.3% (Table 2). The total cumulative duration of exposure to any statins were 165,083 years. The proportion of patients who were exposed to fibrate and ezetimibe were 3.9% and 1.5%, respectively.

**Table 1:** Characteristics of the included subjects

Variable	Value
Number of subjects	379,865
Total number of health examinations	2,117,598
Number of health examination per subject	5 [4–6]
Time period between baseline to last health examination per subject (years)	8.96 ± 2.12
Variables at baseline health examination	
Sex (male)	199,786 (52.6)
Age (years)	51.91 ± 9.19
Fasting glucose (mmol/L)	5.02 ± 0.68
Systolic blood pressure, mmHg	125.38 ± 17.55
Body mass index (kg/m^2^)	23.79 ± 2.87
Current smoker	33,840 (8.9)
Alcohol consumption, frequency per week (times)	
< 1	215,773 (56.8)
1–2	123,573 (32.5)
3–4	25,446 (6.7)
≥ 5	15,073 (4.0)
Exercise, days per week (days)	
< 1	215,736 (56.8)
1–4	129,356 (34.1)
≥ 5	34,773 (9.2)
Household income	
Q1, low	109,219 (28.8)
Q2, middle	139,051 (36.6)
Q3, high	131,595 (34.6)

Data are represented as number (%), mean ± standard deviation, or median [interquartile range]

**Table 2:** Number of patients who were exposed to statins and other lipid lowering agents

Medication	Number (%) of patients who had exposure to medication	Cumulative duration (years) of exposure to medication
Any statin	96,182 (25.3)	165,083
Atorvastatin	61,157 (16.1)	78,627
Rosuvastatin	11,720 (3.1)	14,678
Pitavastatin	8010 (2.1)	7691
Pravastatin	8047 (2.1)	8099
Simvastatin	46,773 (12.3)	46,768
Lovastatin	9291 (2.4)	6045
Fluvastatin	3786 (1.0)	3175
Fibrate^a^	14,926 (3.9)	11,617
Ezetimibe	5847 (1.5)	5830

Data are based on time period between baseline and last health examination of each subject

^a^Bezafibrate, ciprofibrate, etofibrate, fenofibrate, and gemfibrozil

### Factors associated with the change in fasting glucose

**Fig. 2:**
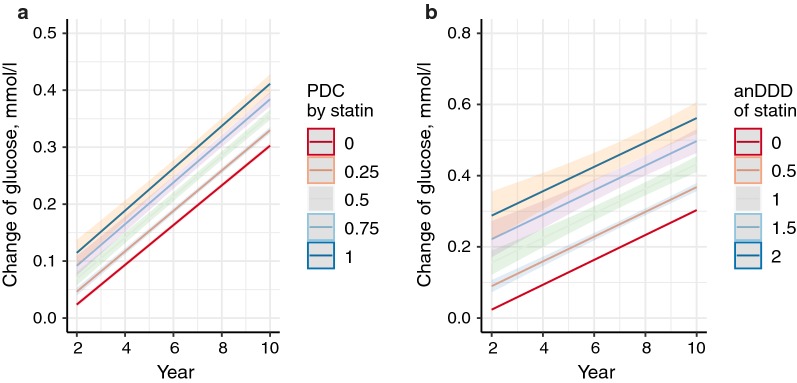
Effects of statin therapy on the change in fasting glucose over time. Plots illustrate the estimated change in fasting glucose (line) and 95% confidence interval (shadow) based on the multivariate linear mixed models adjusted for the variables listed in Table 3. **a** According to adherent use of statin therapy (PDC). **b** According to intensity of statin therapy (anDDD). PDC, proportion of days covered by statins for the time period between baseline and serial measurements of fasting glucose; anDDD, average number of defined daily doses per day for the time period between baseline and serial measurements of fasting glucose

In the multivariate linear mixed model for the change in fasting glucose (Δglu), being male, older age, lower fasting glucose level at baseline, higher systolic blood pressure, higher BMI, being a current smoker, lower household income, higher alcohol consumption, and less exercise were positively associated with Δglu (Table 3). High PDC by statins had a significant positive effect on Δglu (coefficient for PDC 0.093 mmol/L, standard error 0.007, p < 0.001), which indicates that adherent use of statins was associated with an increase in fasting glucose. Unlike statins, PDC by fibrate (coefficient for PDC 0.022 mmol/L, standard error 0.025, p = 0.387) and ezetimibe (coefficient for PDC 0.046 mmol/L, standard error 0.045, p = 0.314) were not significantly associated with Δglu. When we performed analysis including anDDD in place of PDC as a marker of statin intensity, anDDD by statins was also positively associated with Δglu (coefficient for anDDD 0.119 mmol/L, standard error 0.009, p < 0.001). These findings suggested that more adherent and intensive statin therapy could cause an increase in fasting glucose. We plotted the estimated change in Δglu according to statin therapy in the linear mixed models including the interaction term between time and statin therapy (Fig. 2). In the plots, Δglu increased in proportion to PDC and anDDD without the lines crossing over time indicating that there was no interaction effect between time interval and statin therapy.

**Table 3:** Results of the multivariate linear mixed models for the change in fasting glucose level

	Coefficient (β)	Standard error	p
Variables at baseline			
Sex (male)	0.111	0.002	< 0.001
Age (years)	0.003	< 0.001	< 0.001
Baseline fasting glucose (mmol/L)	− 0.764	0.001	< 0.001
Repeatedly measured variables at NHIS health examination			
Systolic blood pressure (per 10 mmHg)	0.028	< 0.001	< 0.001
Body mass index (kg/m^2^)	0.023	< 0.001	< 0.001
Current smoker	0.021	0.002	< 0.001
Alcohol consumption, frequency per week (times)			
< 1	Ref		
1–2	0.034	0.002	< 0.001
3–4	0.094	0.002	< 0.001
≥ 5	0.116	0.003	< 0.001
Exercise, days per week (days)			
< 1	Ref		
1–4	− 0.005	0.001	< 0.001
≥ 5	− 0.011	0.002	< 0.001
Household income			
Q1, low	Ref		
Q2, middle	− 0.004	0.002	0.015
Q3, high	− 0.011	0.002	< 0.001
Time period from baseline to each health examination (year)	0.035	< 0.001	< 0.001
PDC by any statin^a^	0.093	0.007	< 0.001
PDC by fibrate^b^	0.022	0.025	0.387
PDC by ezetimibe	0.046	0.045	0.314
anDDD by any statin^c^	0.119	0.009	< 0.001

Data are derived from the linear mixed models for the change in fasting glucose (mmol/L). These variables were included as fixed-effect variables

*PDC* proportion of days covered by statins during the time period from baseline to serial health examination (ranged from 0 to 1, treated as a continuous variable)

^a^Value is the estimated change in fasting glucose when whole time period is covered by statins (PDC = 1)

^b^Bezafibrate, ciprofibrate, etofibrate, fenofibrate, and gemfibrozil

^c^When ‘anDDD by any statin’ were included in the multivariate linear mixed model instead of ‘PDC by any statin’

### Effect of each statin type on the change in fasting glucose

**Fig. 3:**
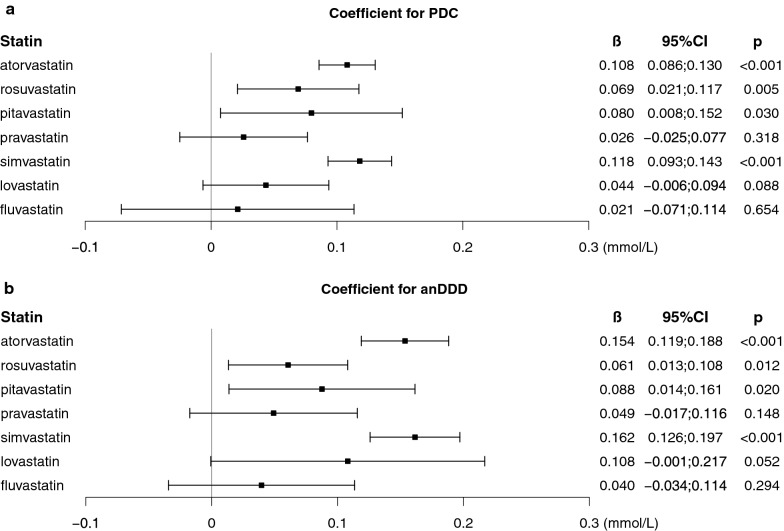
Effects of individual statins on the change in fasting glucose. Data are coefficients (β) and 95% CIs of individual statins on the change in fasting glucose derived by the multivariate linear-mixed models adjusted for the variables listed in Table 3. PDC, proportion of days covered by statins for the period between baseline and serial measurements of fasting glucose; anDDD, average number of defined daily doses per day; *CI* confidence interval

To investigate the effect of each statin type on fasting glucose, we reconstructed the linear mixed model to include PDC for each type of statin. Figure 3 shows the coefficient of PDC for each statin for Δglu. PDC of atorvastatin, rosuvastatin, pitavastatin, and simvastatin had significant positive effects on Δglu. PDC of pravastatin, lovastatin, and fluvastatin also had positive effects on Δglu, but were not statistically significant. When we included anDDD of each statin instead of PDC, there was a similar finding; anDDD by atorvastatin, rosuvastatin, pitavastatin, and simvastatin had significant positive effects on Δglu, but anDDD by pravastatin, lovastatin, and fluvastatin had non-significant positive effects. No statin type had significant interaction effect with time interval in the linear mixed models.

## Discussion

In this longitudinal study using the repeated measurements of fasting glucose level in nationwide health examinations, there was a statistically significant association between statin therapy and increase in fasting glucose in non-diabetic individuals (more adherent and intensive statin users had higher fasting glucose). Non-statin lipid lowering medications of fibrate and ezetimibe were not associated with change in fasting glucose. When we analyzed individual statin types, all types of statins were positively associated with an increase in fasting glucose although the effects of pravastatin, lovastatin, and fluvastatin were not significant. Our findings are in line with the current consensus that statin can induce insulin resistance, hyperglycemia, and new-onset DM [17, 18]. However, our study should not be interpreted as to merely avoid statins due to the hyperglycemic effect. Overall evidence show that the benefits of statins far outweigh the potential hazards [19–21]. Statin therapy was associated with less good glycemic control in diabetes and pre-diabetes, but there was a much lower risk of major cardiovascular events [22, 23]. Real-world studies consistently indicate that statins are frequently suboptimal and under prescribed in populations with high cardiovascular risk [24–27].

When we plotted estimated change in fasting glucose over time (Fig. 2), more adherent and intensive use of statins had positive effects on the change in fasting glucose level, but there was no significant interaction between statins and time interval. That means that use of statins may induce an increase in fasting glucose, but the slope of increase in fasting glucose over time was not associated with the statin therapy. Therefore, we supposed that there is no reason to avoid statins for high risk cardiovascular patients due to the concern for additional hyperglycemia over time induced by long-term statin therapy. This finding was consistent with a prior meta-analysis evaluating the change in hemoglobin A1c (HbA1c) by statins in DM patients; there was no significant interaction between the duration of statin treatment and increase of HbA1c [28]. In addition, we found that the increase in fasting glucose was influenced by an unhealthy lifestyle of smoking, higher alcohol consumption, and lower physical activity. Higher systolic blood pressure, higher BMI, and lower household income were also associated with increase in fasting glucose. These findings support the current recommendations highlighting the importance of lifestyle modifications to prevent impaired glucose tolerance and DM [29].

With expanded indications for statin in the recent guidelines, statins have become the first-line therapy for cardiovascular protection [30]. As more patients take statins, there is awareness of unwanted side effects as well as benefits of statins [31]. Due to there are pathophysiologic and epidemiologic data for a linkage between hyperglycemia and increased cardiovascular or all-cause mortality in diabetic and non-diabetic patients, physicians should be cautious about the potential risk of statin-mediated hyperglycemia and new-onset DM [32, 33]. To maintain the cardiovascular benefit and minimalize the potentially adverse hyperglycemic effect with statins, multi-strategy approach should be considered, including monitoring of fasting glucose, control of predisposing factors to glucose intolerance and unhealthy life-style, proper selection of the statin (type and dosage) and concomitant medications [21, 34]. Screening and risk stratification for diabetes on the initiation of statins and periodical monitoring of fasting glucose would be helpful for early diagnosis and proper glucose control in patients on statin therapy [33, 35]. Sustainable lifestyle changes can improve control of both glucose metabolism and cardiovascular risk [36]. Encouraging healthy dietary pattern, physical activity, body weight control, and quitting smoking could be effective to prevent impaired glucose tolerance and DM [29, 37]. There are a number of medications with diabetogenic property such as thiazide diuretics and steroids; concomitant use of the medications may further increase the risk of impaired glucose metabolism and DM [38, 39].

To explain the diabetogenic effect of statins, numerous mechanisms have been proposed. Glucose and lipid metabolism are interconnected in many ways [40]. Gene variants involved in lipid metabolism (Niemann-Pick C1-Like 1, Proprotein convertase subtilisin/kexin type 9, and HMGCR) are significantly associated with obesity, hyperglycemia and DM [41–43]. Lower prevalence of type 2 DM in patients with familial hypercholesterolemia suggests genetic links between glucose and lipid metabolism [44]. Statins-induced cholesterol-dependent conformational changes in glucose transporter (GLUT) proteins can lead to impaired glucose uptake in the cells and insulin resistance [45]. Inhibition of de-novo cholesterol synthesis by statins results in deleterious inflammation and oxidation within islet β-cells, which lead to cellular apoptosis and impaired insulin secretion [46]. Biosynthesis of the isoprenoid side chain of coenzyme Q10 is suppressed by statins, which has been implicated in the downregulation of GLUT4 synthesis, mitochondrial oxidative stress, delayed adenosine triphosphate production, and apoptosis of β-cells, causing impaired glucose-stimulated insulin secretion [19, 47, 48]. Simvastatin and atorvastatin have been shown to reduce the level of adiponectin, which is a hormone with anti-inflammatory and anti-diabetogenic properties secreted by adipocytes [19, 49]. One of the most common complaints with statins is muscle-related symptoms (muscle pain, myopathy, myalgia, and fatigue) [50]. Statin-related muscle complaints are frequently exacerbated by exercise, which further limits physical activity and exercise performance [51]. Since physical inactivity is associated with abdominal adiposity, body weight gain, and glucose intolerance, limited physical activity by statins may be a mediator of glucose intolerance [23, 52, 53].

There are scarce data on the effects of individual statins on DM and diabetogenic potency. Previous meta-analyses with randomized and observational studies have shown no clear difference between statin types in terms of DM incidence [5, 54]. The Women’s Health Initiative showed that an increased risk of DM was observed for all types of statins [55]. Therefore, all statins seem to have a diabetogenic potency considered as a medication class effect [17]. However, individual statins have different mechanisms of action and pharmacokinetic properties, and experimental data suggest that different statins have varying effects on glucose metabolism [56]. Based on available clinical and experimental data, it is generally accepted that atorvastatin, rosuvastatin, simvastatin, lovastatin, and fluvastatin have an unfavorable influence on glycemic parameters and risk of new-onset DM [57].

Some data suggested that pravastatin may have relatively weak diabetogenicity compared to that of other statins [3]. In a network meta-analysis, pravastatin had the lowest risk of inducing new-onset DM among the high-dose statin therapy (atorvastatin 80 mg, rosuvastatin 20 mg, simvastatin 40 mg, and pravastatin 40 mg), but the difference was not statistically significant [58]. The West of Scotland Coronary Prevention Study showed that pravastatin therapy resulted in a 30% reduction in the risk of new-onset DM compared to placebo [59]. The relatively lower risk of DM with pravastatin was also reported in retrospective studies using the real-world data [60, 61]. Unlike other statins, pravastatin may increase plasma adiponectin levels and improve insulin sensitivity mediated by the elevation of calcitriol [62]. The hydrophilicity of pravastatin may have little effect on membrane-embedded proteins involving glucose metabolism such as the GLUT [63]. However, the Prospective Study of Pravastatin in the Elderly at Risk (PROSPER) trial showed that the risk of new-onset diabetes increased by 30% in patients treated with pravastatin compared with those given a placebo [64].

Pitavastatin is the newest statin and has been shown to be well-tolerated with fewer side effects and a relatively low drug interaction profile [65]. A recent meta-analysis of randomized controlled trials with pitavastatin suggested that pitavastatin did not adversely affect glucose metabolism or increase risk of developing diabetes compared to the controls [66]. In the Japan Prevention Trial of Diabetes by Pitavastatin in Patients With Impaired Glucose Tolerance (J-PREDICT) trial, treatment with pitavastatin reduced the risk of new‐onset DM by 18% in patients with impaired glucose tolerance [67]. The Japanese long-term prospective post-marketing surveillance LIVALO Effectiveness and Safety (LIVES) study demonstrated that treatment with pitavastatin lowered HbA1c (from 8.1% at baseline to 7.4% at 6 months) in the patients with poorly controlled diabetes [68].

In our study, all classes of statins were associated with an increase in fasting glucose although the effects of pravastatin, lovastatin, and fluvastatin were not significant. In our data, the hyperglycemic effects of pitavastatin does not seem to be lower than that of other statins in contrast to the favorable reports for pitavastatin on glucose metabolism. A recent retrospective cohort study in Korea also reported that pitavastatin had the highest risk of new-onset DM compared to other statins [69].

In a number of prior studies, there are inconsistent findings about the diabetogenic property of individual statins. The discrepancy could be originated from difference in baseline characteristics of study population, coexisting risk factors, follow-up duration, medication adherence and so on. It is known that statin-induced DM risk is higher in pre-diabetes and patients with predisposing factors for DM such as old age and obesity [34]. The short-term and long-term effects on glucose metabolism may be different in accordance with type of statins and underlying characteristics of patients [70]. The diabetogenic effect of statins may be varied in genetic background [71]. In patients who were prescribed statins, discontinuation and poor adherence were common in clinical practice, which limits assessing the diabetogenic effect of statins [72]. The effect of statins on glucose metabolism are complex and interconnected by multiple pathways; individual statins may have a heterogenous impact on the multiple processes [11]. To obtain further knowledge of individual statins on glucose metabolism, there is need for further extensive studies.

This study had both strengths and limitations. From a population-based nationwide health screening program, we collected data from a large cohort of 379,865 patients over a 10-year study period. Serial data of more than 2,000,000 measurements of fasting glucose were analyzed. Besides data on fasting glucose, we also included detailed data on blood pressure, BMI, and lifestyle. The significant effects of an unhealthy lifestyle on fasting glucose including smoking, higher alcohol intake, higher BMI and lower physical activity may support the relevance of this study. In Korea, statins must be prescribed by physicians and refill programs at pharmacies are not allowed. Using the health insurance claim data, prescription records for individual statin could be accessed. However, the actual intake of statins in subjects might be different from the prescription records. Fortunately, there was good correlation between prescription and real exposure to drugs in prior studies [73, 74]. Our study was retrospectively performed and due to the observational nature, there was a possibility of hidden bias due to uncollected data. We excluded a large proportion of subjects with DM or who had received anti-diabetic medication during the study period to eliminate the potential bias caused by the effects of this medication on glucose level. The item for physical activity lacked data for the type and duration of exercise. This study was based on Koreans; the response to statins might be varied in other genetic population. Therefore, our results should be interpreted with caution and further studies are needed to confirm these findings in other populations.

## Conclusions

There was significant increase of fasting glucose in non-diabetic individuals in proportion to adherent and intensive use of statins. Among the statin subtypes, use of atorvastatin, rosuvastatin, pitavastatin and simvastatin were associated with significant increase in fasting glucose. Pravastatin, lovastatin, and fluvastatin also had trend toward an increased fasting glucose, but were statistically non-significant. These finding suggested medication class effect of all types of statins predisposing hyperglycemia although there was some difference in the degree according to the type. We need to find optimal strategy to maximize the cardiovascular benefit and minimalize the hyperglycemic effect by statins.

## Abbreviations

anDDD: average number of defined daily doses per day
BMI: body mass index
CI: confidence interval
DDD: defined daily doses
DM: diabetes mellitus
GLUT: glucose transporter
HbA1c: hemoglobin A1c
HMGCR: 3-hydroxy-3-methylglutaryl-CoA reductase
ICD-10: International Statistical Classification of Diseases and Related Health Problems 10th Revision
NHIS: National Health Insurance Service
NHIS-HEALS: National Health Insurance Service-National Health Screening Cohort
PDC: proportion of days covered
Δglu: changes in fasting glucose
